# A Novel Algorithm to Estimate the Significance Level of a Feature Interaction Using the Extreme Gradient Boosting Machine

**DOI:** 10.3390/ijerph19042338

**Published:** 2022-02-18

**Authors:** Chao-Yu Guo, Ke-Hao Chang

**Affiliations:** Division of Biostatistics, Institute of Public Health, School of Medicine, National Yang Ming Chiao Tung University, Taipei 112, Taiwan; khchang.hchs@gmail.com

**Keywords:** interaction, machine learning, cross-validation, XGB

## Abstract

Recent studies have revealed the importance of the interaction effect in cardiac research. An analysis would lead to an erroneous conclusion when the approach failed to tackle a significant interaction. Regression models deal with interaction by adding the product of the two interactive variables. Thus, statistical methods could evaluate the significance and contribution of the interaction term. However, machine learning strategies could not provide the *p*-value of specific feature interaction. Therefore, we propose a novel machine learning algorithm to assess the *p*-value of a feature interaction, named the extreme gradient boosting machine for feature interaction (XGB-FI). The first step incorporates the concept of statistical methodology by stratifying the original data into four subgroups according to the two interactive features. The second step builds four XGB machines with cross-validation techniques to avoid overfitting. The third step calculates a newly defined feature interaction ratio (FIR) for all possible combinations of predictors. Finally, we calculate the empirical *p*-value according to the FIR distribution. Computer simulation studies compared the XGB-FI with the multiple regression model with an interaction term. The results showed that the type I error of XGB-FI is valid under the nominal level of 0.05 when there is no interaction effect. The power of XGB-FI is consistently higher than the multiple regression model in all scenarios we examined. In conclusion, the new machine learning algorithm outperforms the conventional statistical model when searching for an interaction.

## 1. Introduction

Recent studies of the interaction effect in cardiac research successfully discovered crucial findings. Electromagnetic interactions between implanted cardioverter defibrillators and left ventricular assist devices were examined [1]. An interaction between arousals and ventilation during Cheyne–Stokes respiration in heart failure patients was reported [2]. Kawashima [3] showed that there was a significant interaction in mortality between treatment effect (percutaneous coronary intervention (PCI) and coronary artery bypass grafting (CABG)) and the presence or absence of heavily calcified lesions (HCLs) (P_interaction_ = 0.005). Another study [4] estimated the risk of death related to ventricular arrhythmia in time-updated models. This study examined the interaction between heart failure etiology and the effect of sacubitril/valsartan. Hazard ratio in patients with an ischemic etiology was 0.93 (0.71–1.21) versus 0.53 (0.37–0.78) in those without an ischemic etiology (p for interaction = 0.020). Therefore, developing a novel algorithm to detect the interaction effect is beneficial for cardiology and various research fields.

Regression analysis detects interaction between two independent variables by the product of the two independent variables $Y=\beta_{0}+\beta_{1}X_{1}+\beta_{2}X_{2}+\beta_{3}X_{1}X_{2}$. Testing whether the slope of the interaction term ($\beta_{3}$) is equal to zero determines the significance of this interaction term. When the *p*-value is less than the significance level of 0.05, we declare that the interaction is significant [5].

Compared with conventional statistical approaches, machine learning algorithms improve the predictive accuracy by fitting the model with a tremendous training process [6]. Driven by big data, a machine learns if its performance at tasks improves with experience [7]. When building a model using machine learning techniques, the first step is to stratify the data into two independent subsets: the training and testing sets. The training data create datasets only used to train the hyperparameters. As a result, the optimized model parameters are estimated based on the training data. The testing data are not included in the training process but only evaluate the trained machine or model. To avoid overfitting, the K-fold cross-validation (CV) is the ideal solution [8,9], where the CV error could be measured by accuracy, mean square error (MSE), and F1 Score [10].

Among the learning algorithms, there are unsupervised and supervised learning. Unsupervised machine learning only examines the associations between a set of predictors [7], for which the principal components analysis (PCA) [11] is the most popular method. In contrast, supervised machine learning deals with a dependent variable or the outcome of interest. For supervised learning, regression for a continuous outcome variable and classification for a categorical outcome are carried out through three primary applications—the generalized linear model (GLM) [12], the logistic regression model [13], and the support vector machine (SVM) [14]. 

In addition to the SVM, tree-based designs are the most popular due to simple applications and interpretations. Random forest could provide feature importance through decision trees [15]. The algorithm of decision trees was examined in 2001 by Breiman [16], showing that a type of ensemble method is a collection of multiple weak classifiers to produce a robust classifier [17]. Classification and regression tree (CART) is a decision tree for predictive classification and continuous value [18] that adopts the binary division rule. There are only two branches each time a division is generated, and the Gini index could determine which branch is the best. The random forest model is a popular and powerful tool for classification problems [16].

Instead of using the bagging technique in random forest models, the gradient boosted decision tree (GBDT) [19] builds one decision tree at a time to fit the residual of the trees that precede it to increase the predictive ability. The extreme gradient boosting (XGB) machine [20] extends the GBDT and allows more hyperparameters that regulate the effect estimates and produce incredible predictive power. 

Recent research (Wright, Ziegler, and König [21]) suggested that the tree model could not pick two interaction variables (features) at the beginning in simulating multiple different interactions. It is an internal node, especially when the marginal effect of the interaction variable is small. Although the tree model can handle partial interaction effect, such modification is easily affected by the marginal effect. In addition, a random forest is composed of many decision trees. When building the trees, the random forest only selects some variables as the nodes. As a result, each tree may not include the interaction variables, and the prediction could be biased.

Since the tree-based design has the most intuitive structure to deal with interactions, this research aims to develop an algorithm to evaluate the statistical significance of a feature interaction by modifying the data structure of the XGB machine. The new approach is named the extreme gradient boosting machine for feature interaction (XGB-FI).

## 2. Materials and Methods

The training and testing sets contain 80% and 20% of the data, respectively. Assuming there is only a single two-way interaction, the XGB-FI algorithm has five steps with 10-fold cross-validation to avoid overfitting:

1. Build the XGB machine using the original dataset without the two interactive features (noted as ${\mathrm{XGB}}^{-X1-X2}$) and obtain the root-mean-squared error (RMSE);
2. Stratify the original data into four subgroups according to the two interactive features;
3. Build separate XGB machines for each of the four stratified subsets and then average the four RMSEs (noted as ${\mathrm{XGB}}_{+4\;stratum}$);
4. Calculate a newly defined feature interaction ratio (FIR) for all possible combinations of predictors as $\mathrm{FIR}=\frac{\;Mean\;\mathrm{RMSE}\;of\;{\mathrm{XGB}}_{+4\;stratum}}{\mathrm{RMSE}\;of\;{\mathrm{XGB}}^{-X1-X2}}$;
5. Declare significance or obtain the empirical *p*-value using the threshold $\left(Q_{1}-1.5\times IQR\right)$, where *Q*_1_ is the first quartile (25th percentile), and *IQR* is the interquartile range, according to the empirical FIR distribution.

It is worth noting that the above algorithm adopts the same hyperparameter of the XGB by grid search using the whole data with every feature. The learning rate is 0.05. The number of trees is 300. The early stopping round is 10. The maximum depth of a tree is 6. The rest hyperparameters are default settings.

In the first step, the XGB machine is built using the original dataset without the two interactive features. We denoted the model as ${\mathrm{XGB}}^{-X1-X2}$. This model estimates the interaction effect’s impact since the XGB machine failed to incorporate the two variables and the interaction term in the analysis.

**Figure 1:**
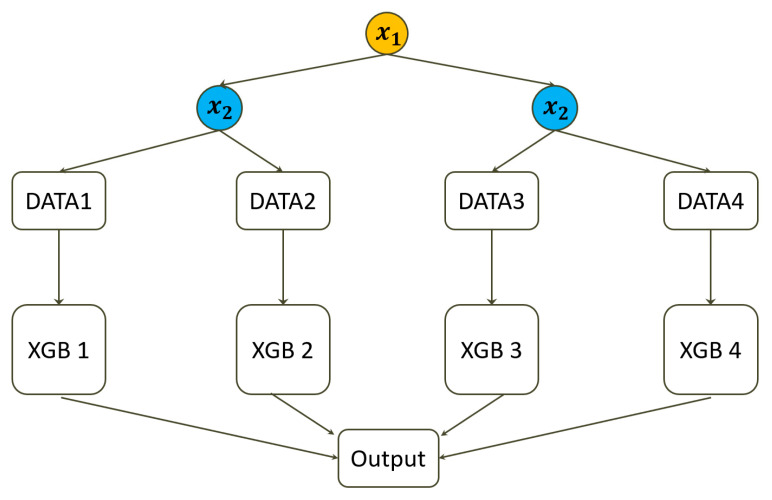
The data structure of the XGB-FI.

In the second step, a concept of stratification is adopted that eliminates the impact of interactions [22]. Assuming the two interactive predictors are dichotomous, the original data are stratified into four subsets. If one or two predictors are continuous, the median is the threshold to dichotomize the predictor. In this way, there is no need to consider whether the distribution is normal or symmetric. Other than the median, one could consider the mean, the first or third quartile, or any arbitrary percentile to dichotomize the predictors. As a result, Figure 1 displays the data structure of the XGB-FI, and the (XGB 1) to (XGB 4) are separately fitted to the four subsets with the same hyperparameters.

Note:

$$\mathrm{XGB}\;1:\;X_{1}\geq median\;and\;X_{2}\geq median$$

$$\mathrm{XGB}\;2:\;X_{1}\geq median\;and\;X_{2}<median$$

$$\mathrm{XGB}\;3:\;X_{1}<median\;and\;X_{2}\geq median$$

$$\mathrm{XGB}\;4:\;X_{1}<median\;and\;X_{2}<median$$

In the third step, we implement another four XGB machines within each stratum and then derive the mean of the four RMSEs. It is worth noting that the four subsets do not contain the two variables with feature interaction (*X*_1_ and *X*_2_.). We denoted this model as ${\mathrm{XGB}}_{+4\;stratum}$. The result of this model contains the improvement or gain by removing the interaction effect but not the main effect of the two interactive variables.

In the fourth step, we define the feature interaction ratio (FIR) as

$$\;\mathrm{FIR}=\frac{\;Mean\;\mathrm{RMSE}\;of{\;\mathrm{XGB}}_{+4\;stratum}}{\mathrm{RMSE}\;of{\;\mathrm{XGB}}^{-X1-X2}}$$

The denominator of the FIR represents the RMSE not controlling for interaction and the main effect of *X*_1_ and *X*_2_. In contrast, the numerator is correctly adjusted for feature interaction but not the main effect. As a result, the difference between the numerator and the denominator is only the interaction effect.

As the numerator represents the gain of treating the interaction impact, the smaller FIR indicates a more substantial interaction effect. This step calculates numerous FIRs for all possible combinations of interactions. If the total number of predictors is *K*, there are $C_{2}^{K}=\frac{K!}{2!\left(K-2\right)!}$ combinations.

The last step declare statistical significance with the threshold: ($Q_{1}-1.5\times IQR$), where *Q*_1_ is the first quartile (25th percentile), and *IQR* is the interquartile range, according to the empirical FIR distribution of $C_{2}^{K}=\frac{K!}{2!\left(K-2\right)!}$ combinations. Suppose the interaction term of the particular interest leads the FIR below the threshold. In that case, we declare that the feature interaction is statistically significant, which means that it is unlikely to occur by chance. The percentile of FIR seems to estimate the empirical *p*-value for the feature interaction. However, we discovered that the 95th percentile obtained from the empirical distribution did not provide the correct significance level. Therefore, we used a nonparametric threshold for outliers in the box plot.

### Simulation Study

The computer simulation was conducted by Python version 3.7.7. We used the “multivariate_normal function” in the “NumPy” package to generate six correlated variables, then the “normal function” generated 10 independent noise variables. The covariance matrix determines the relationship between Y and the 15 predictors ($X_{1},\cdots ,\;X_{15}$). Figure 1 shows the Heat map of the 16 variables. 

**Figure 2:**
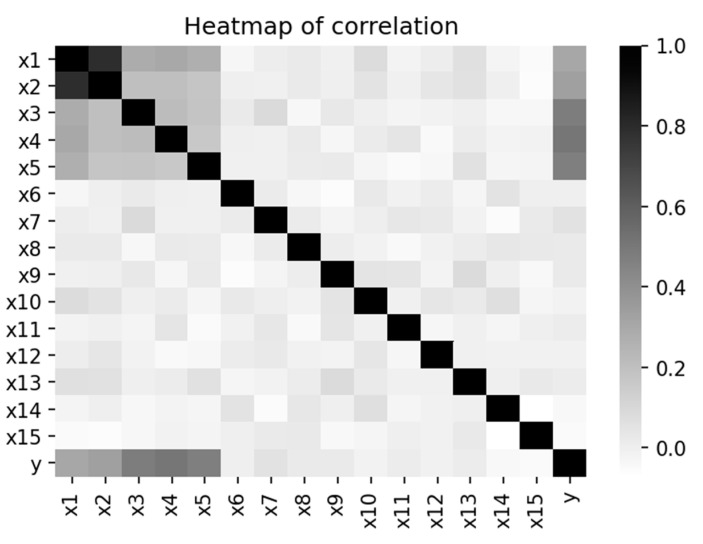
Correlation structure of simulated data.

The correlation coefficient between the two interactive features ($X_{1}$ and $X_{2}$) is 0.8. Both $X_{1}$ and $X_{2}$ have a correlation coefficient of 0.3 with the outcome variable Y. Marginal effects ($X_{3}$, $X_{4}$ and $X_{5}$) have a correlation coefficient of 0.5 with Y, and the correlation among the three marginal effects is 0.2. Therefore, ($X_{3}$, $X_{4}$ and $X_{5}$) has a higher impact on Y than ($X_{1}$ and $X_{2}$). Correlation among the noises ($X_{6}$ to $X_{15}$) is zero, and they are uncorrelated with any variable in the dataset, including the outcome variable Y. Figure 2 is the heatmap of the correlation coefficients that displays the correlation structure of simulated data. Data management and analysis tools used the “pandas” and “scikit-learn” packages. The samples sizes are 500 and 1000. Since there are 15 predictors, the fourth step created a total of $C_{2}^{15}=\frac{15!}{2!13!}=105$ FIRs.

Notably, the simulated data have no interaction effect, although $X_{1}$, $X_{2}$, and Y are correlated. The interaction effect is added when both the values of $X_{1}$ and $X_{2}$ are higher than their medians. The interaction effect has three different settings. The first setting assumes a mild interaction effect. Thus, the interaction is randomly assigned to individuals according to the normal distribution with the mean 0.5 and variance 1. We added normal (1,1) for a moderate effect, and a considerable interaction has the extra value of normal (2,1). 

In addition to the correlation structure in Figure 2, we conducted more scenarios to examine the impact of marginal effects. Table 1 shows different correlation structures between Y and the predictors. We aimed to determine whether the magnitude of association between the marginal effects ($X_{3},\;X_{4},\;X_{5})$ and the outcome Y would modify the performance of the two interactive features ($X_{1}$, $X_{2}$).

## 3. Results

**Figure 3:**
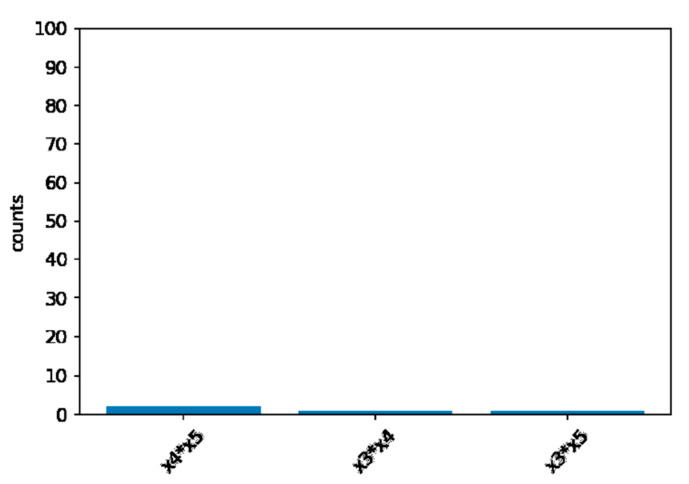
The null distribution of the XGB-FI.

Computer simulation has 100 repetitions for each scenario. Under the null hypothesis of no interaction between $X_{1}$ and $X_{2}$, the FIR never exceed the threshold of ($Q_{1}-1.5\times IQR$). In Figure 3, we can see that only $X_{4}$ and $X_{5}$, $X_{3}$ and $X_{4}$, and $X_{3}$ and $X_{5}$ have reached the threshold. Although too conservative, the XGB-FI demonstrated a valid type-I error under the 5% significance level.

**Figure 4:**
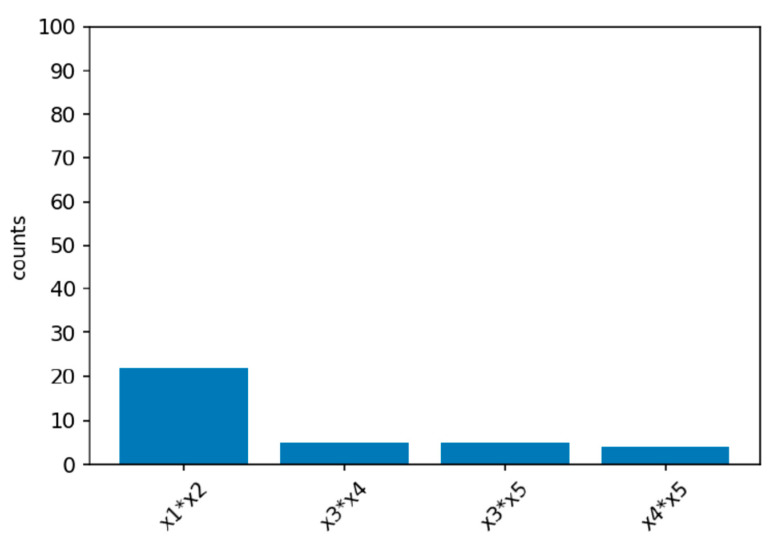
Power simulation under the mild interaction effect of normal (0.5, 1) for XGB-FI.

**Figure 5:**
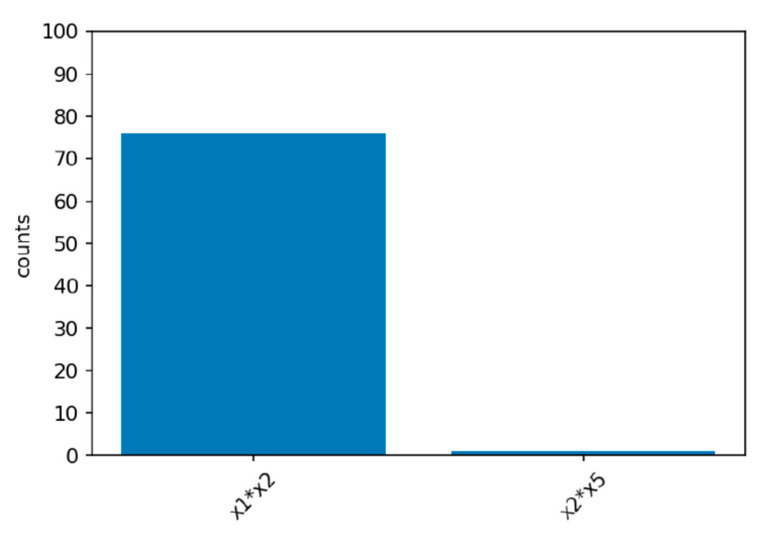
Power simulation under the moderate interaction effect of normal (1, 1) for XGB-FI.

**Figure 6:**
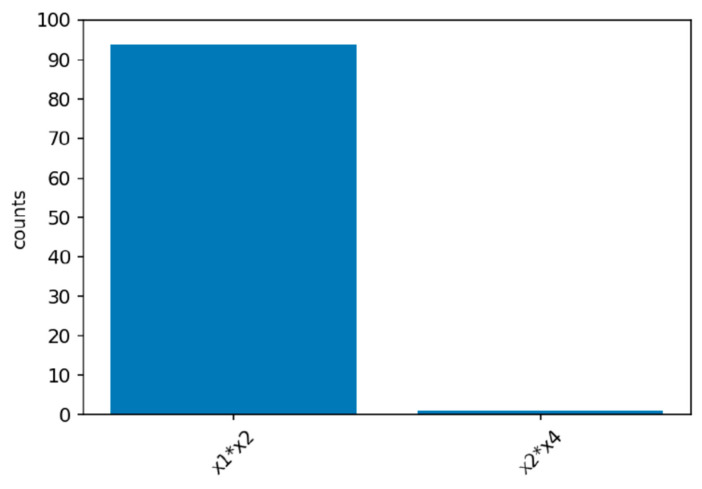
Power simulation under the considerable interaction effect of normal (2, 1) for XGB-FI.

Power simulations for the XGB-FI with a sample size of 1000 are displayed in Figure 4, Figure 5 and Figure 6. The first bar in the horizontal axis represents the power of detecting feature interaction between $X_{1}$ and $X_{2}$. Other bars are type-I errors since there were no interactions for other predictors. With a sample size of 500 (1000), the statistical power of the XGB-FI is 14% (23%), 62% (77%), and 79% (94%) for the mild, moderate, and considerable interaction effects. The results revealed that statistical power increases when the interaction effect becomes more significant. In addition, when the sample size increases from 500 to 1000, statistical power rises accordingly. The power comparisons of multiple regression are added in Table 2 and Table 3 with different sample sizes. The 95% confidence interval for each scenario is displayed in parenthesis. The XGB-FI consistently has higher statistical power to identify feature interaction than the regression models.

From Table 4, it can be inferred that the XGB-FI is consistently more powerful in all scenarios. Although the magnitude of association between the marginal effects ($X_{3},X_{4},X_{5})$ and the outcome Y changes the power of the two interactive features ($X_{1}$, $X_{2}$), the superiority of the XGB-FI is consistent. Regarding the type-I error simulations, the FIR of ($X_{1}$, $X_{2}$) does not show any significant result in 100 repetitions. Although the estimated Type-I error is too conservative, it is a valid test for identifying the interaction effect.

## 4. Discussion

In this research, we proposed a novel algorithm to assess the significance level of a feature interaction based on a modified structure of the XGB machine. In addition, we defined a new feature interaction ratio (FIR). We also explicitly indicated the threshold to declare significance based on the empirical distribution of all null interactions in the set of predictors. Computer simulations confirm that the XGB-FI has a valid type-I error rate under the null hypothesis of no interaction effect. Most importantly, the XGB-FI outperforms the conventional regression model in detecting the interaction effect.

The limitation of XGB-FI is that at least 15 predictors are required to generate the null distribution of FIR for all possible interactions. If the data contain less than 15 features, then the combination of potential interactions is less than 105, and the probability of declaring significance drops accordingly. More predictors would result in a more reliable null distribution of the FIRs and assure the statistical power of the XGB-FI. Although the simulations assume continuous features, scenarios for categorical or ordinal feature interactions are intuitive.

This research focused on a two-way feature interaction. However, a higher-order interaction such as a three-way interaction could adopt similar concepts. Future studies could carry out the empirical distribution of the FIRs and declare significance or assess the *p*-value under a higher-order interaction effect.

Similar to a previous study [23], simulations were conducted with 100 repetitions for each situation. More repetitions could obtain more accurate estimates. However, all scenarios yielded zero type-I error estimates. In addition, the power of XGB-FI is much higher than the multiple regression. The 95% confidence interval of the empirical power estimates for the XGB-FI is significantly different from that of the multiple regression. Therefore, more repetitions would not alter the scientific discoveries, and 100 repetitions are satisfactory in this study.

In computer simulations, we adopted a similar approach in our recent research [24] and generated data according to the multivariate normal distribution. A variance–covariance matrix describes the correlation of the predictor and outcome variables. However, there are numerous ways of simulating datasets. For example, the outcome variable could be generated by a function of predictors [25]. In this way, a more complicated structure for feature interaction could be assessed, which would be a prudent topic for future research.

Although this study used simulated data, the XGB-FI could be implemented in cardiac research or other fields. The discovery of more complicated interaction effects will benefit tremendous clinical applications.

The limitation of this research is that we only simulated continuous predictors and outcomes. However, feature interaction is a very complex and nuanced area of study. There are other combinations with nominal and ordinal variables that introduce feature interaction, the nature of the interaction, the localization of the interaction effect, or the range of effect. Therefore, the XGB-FI may not be the optimal strategy without considerable research, and a future study is highly desired.

## Figures and Tables

**Table 1:** Different correlation structures between Y and the predictors.

	Correlation Coefficient Corresponding to Y	
	$X_{1},\;X_{2}$	$X_{3},\;X_{4},\;X_{5}$
Scenario 1	0.3	0.3
Scenario 2	0.5	0.5
Scenario 3	0.5	0.8
Scenario 4	0.8	0.8

**Table 2:** Power comparisons between the XGB-FI and multiple regression with sample size 500.

	Interaction Effect		
	Mild	Moderate	Considerable
XGB-FI	0.14 (0.07, 0.21)	0.62 (0.52, 0.72)	0.79 (0.71, 0.87)
Multiple regression	0.04 (0.002, 0.078)	0.20 (0.12, 0.28)	0.52 (0.42, 0.62)

**Table 3:** Power comparisons between the XGB-FI and multiple regression with sample size 1000.

	Interaction Effect		
	Mild	Moderate	Considerable
XGB-FI	0.23 (0.15, 0.31)	0.77 (0.69, 0.85)	0.94 (0.89, 0.99)
Multiple regression	0.12 (0.06, 0.18)	0.41 (0.31, 0.51)	0.85 (0.78, 0.92)

**Table 4:** Power comparison under Scenarios 1 to 4.

		Magnitude of Interaction Effects		
Scenarios		Mild	Moderate	Considerable
1	XGB-FI	0.94	1	1
	Multiple regression	0.04	0.37	0.69
2	XGB-FI	0.95	1	0.98
	Multiple regression	0.09	0.43	0.88
3	XGB-FI	0.23	0.8	0.96
	Multiple regression	0.1	0.4	0.88
4	XGB-FI	1	1	0.95
	Multiple regression	0.12	0.35	0.85

## Data Availability

Simulated data only.
